# Insights into artificial waterhole utilization patterns by elephants and rhinos: Lessons from a South African Nature Reserve

**DOI:** 10.1371/journal.pone.0312158

**Published:** 2024-10-16

**Authors:** Eilidh Smith, Leslie Robert Brown, Alan Sean Barrett

**Affiliations:** Applied Behavioural Ecology and Ecosystem Research Unit, Department of Environmental Sciences, UNISA Science Campus, Florida, Republic of South Africa; Institute of Zoology, CHINA

## Abstract

Artificial water provisioning is a common practice in southern African nature reserves, where different game species exhibit preferences for specific waterhole types. The movement patterns and behaviour of elephants and rhinos are closely linked to water availability, with these mega-herbivores noticeably influencing the environment and other species they interact with at waterholes. Since there is limited research on this topic, understanding preferences for different types of artificial waterholes is crucial, particularly during periods of water scarcity. This knowledge enables reserve managers to effectively manage the numbers and types of waterholes. In this study, we investigate artificial waterhole selection and preferences by elephants and rhinos in the Olifants West Nature Reserve, South Africa. The study area featured various waterhole types, including earth dams, concrete pans, reservoirs, and troughs. By employing camera traps, we analysed visitation frequency, timing of visits, and factors influencing visit frequency. Our findings revealed distinct preferences for specific waterhole types among different social groupings of the study species. Breeding herds of elephants predominantly utilized reservoirs and occasionally visited troughs, while bachelor herds favoured earth dams. Black rhinos showed a preference for earth dams, whereas white rhinos selected troughs and earth dams, with bachelor groups favouring troughs and female rhinos favouring earth dams. The outcomes of this study have significant implications for the development of comprehensive conservation plans in areas where these species reside, and for potential release sites.

## Introduction

Artificial waterholes are widely used in nature reserves and their importance in sustaining wildlife during dry seasons and droughts is often a subject of debate [1, 2]. These waterholes have a significant impact on the environment and to the animal species that depend on them [3]. Herbivores’ water requirements influence their movement patterns, behaviour and preferences for certain waterhole types (both natural and artificial) based on the morphology of these waterholes [4–7]. This, in turn, affects vegetation composition, biodiversity, and animal interactions at water sources [3, 7–13]. The presence of artificial waterholes often leads to the congregation of animals that would typically rely on natural water sources, resulting in interspecific and intraspecific competition for water, particularly during periods of scarcity [8].

The design of artificial waterholes, such as high-sided reservoirs for example, favours certain species like elephants that can access water with their trunks, while excluding smaller animals. Waterholes also vary in shape and lateral extent and may be associated with mud wallows, which are important for thermoregulation and parasite control for species like warthogs (*Phacochoerus africanus*) and rhinos (*Diceros bicornis* and *Ceratotherium simum*) [14–17]. Other factors influencing waterhole selection include perceived predation risk [18, 19], avoidance of competition [20, 21], species behaviour at waterholes [21, 22], faecal bacterial loads [23], and gregariousness of visiting species [24].

Mega herbivores such as elephants (*Loxodonta africana*) and rhinos (*Diceros bicornis* and *Ceratotherium simum*) have pronounced impacts on other species and the environment [13, 15, 25, 26]. Understanding the waterhole preferences and visitation patterns of these species is crucial for their management and relocation, especially considering the severe poaching pressure they face for their horns and tusks [27–32]. Protecting natural environments and safeguarding vulnerable species like elephants and rhinoceroses are top priorities for protected area managers and wildlife departments. Effective management of protected areas requires comprehensive plans to guide conservation efforts and ensure the preservation of habitats and their resident species. Managing natural and artificial water sources is a fundamental aspect of such planning, encompassing the selection, placement, utilization, and impact of artificial waterholes on surrounding vegetation [1–3, 5, 6, 8, 10, 20, 21, 24, 32].

African elephants (*Loxodonta africana*) are mixed feeders that typically drink water daily but can go without it for up to four days [32]. Adult elephants require 150 to 300 litres of water per day [27, 32, 33] and do not have a preferred drinking time [27, 32–34]. In contrast, black rhinoceros (*Diceros bicornis*) are browsers that enjoy wallowing and need to drink water once every four to five days but will drink more frequently (35 litres per day) if water is readily available [32–34]. Adult black rhino prefer artificial waterholes over natural ones and drink throughout the day and night [32–34]. White rhinos (*Ceratotherium simum*), on the other hand, are grazers that need to drink water daily, and like the black rhino, also wallow [27, 32, 34]. An adult wild white rhino requires up to 72 litres of water a day [32–34], shows no preference for a particular waterhole type [32–34], and drinks at least twice a day [32–34], most often during the late afternoon and after dusk (17:00 to 21:00) [27, 32], but also during the morning and midday periods [32, 33].

Given their dependence on water, elephants, white rhinos, and to a lesser extent black rhinos are influenced by water availability [3, 27, 32–34]. Understanding the factors that affect the behaviour and movement patterns of these mega-herbivores is of particular interest from a conservation management perspective, considering their significant impact on the environment, other species, and each other [13, 25, 26]. No comparative regional studies were found that investigated waterhole type preferences among various elephant social groupings. Utilization patterns of waterholes by elephants influence how other wildlife species use these water sources [20, 35]. As natural surface-water availability diminishes during the dry season, the utilization of artificial water sources by water-dependent species exhibits seasonal variations, becoming increasingly important [3, 21, 36].

Unfortunately, elephant and rhino populations are facing severe threats due to poaching, habitat fragmentation, and illegal trade of their products [37, 38]. These large herbivores, especially in water-limited habitats, are particularly vulnerable to the impacts of poaching [39]. The demand for ivory, rhino horn, and related products has resulted in significant declines in elephant and rhino populations across Africa [40]. Combined with habitat fragmentation, illegal harvesting has left small, isolated populations of these species, posing further threats to their survival [41]. Acquiring additional information about the water utilization behaviour of elephants, black rhinos, and white rhinos, including their preferences for specific artificial waterhole types, is crucial for effective management and protection of these animals.

This study focuses on the utilization of four different waterhole types by elephants, black rhinos, and white rhinos in the Olifants West Nature Reserve (OWNR) in southern Africa. The study investigates the timing of their visits to waterholes, the frequency of their visitations during different seasons, and their preferences for specific artificial waterhole types. This knowledge is valuable for managing areas that support populations of elephants, black rhinos, and white rhinos, as well as for establishing new areas for their relocation to enhance population conservation efforts. Investigating the utilization of artificial waterholes by these study species is particularly important considering their declining numbers and the ecological and economic consequences associated with their decline [27–30]. While information on the population densities of these species would contribute to a more comprehensive discussion on waterhole attendance, such data was not provided to the researchers due to the high levels of poaching associated with these species.

Understanding the preferences and utilization patterns of elephants, black rhinos, and white rhinos for artificial waterholes is important for the effective management of these species. This study provides valuable insights into their visitation patterns, preferences for specific waterhole types, and seasonal variations in waterhole utilization. Such information can guide conservation strategies, including the optimization of waterhole design and placement, to reduce competition and support the long-term survival of these iconic and threatened mega-herbivores. The findings will assist conservation managers in making informed decisions to protect these species in both current and future protected areas.

## Methods

The study was conducted by the first author, who was a student employee of the nature reserve where the research took place. The researcher obtained permission from reserve management to do the study, which involved monitoring waterholes. Ethical clearance for the study (2014/CAES/037) was granted by the University of South Africa’s (UNISA) Ethics Department.

### Study site

**Fig 1:**
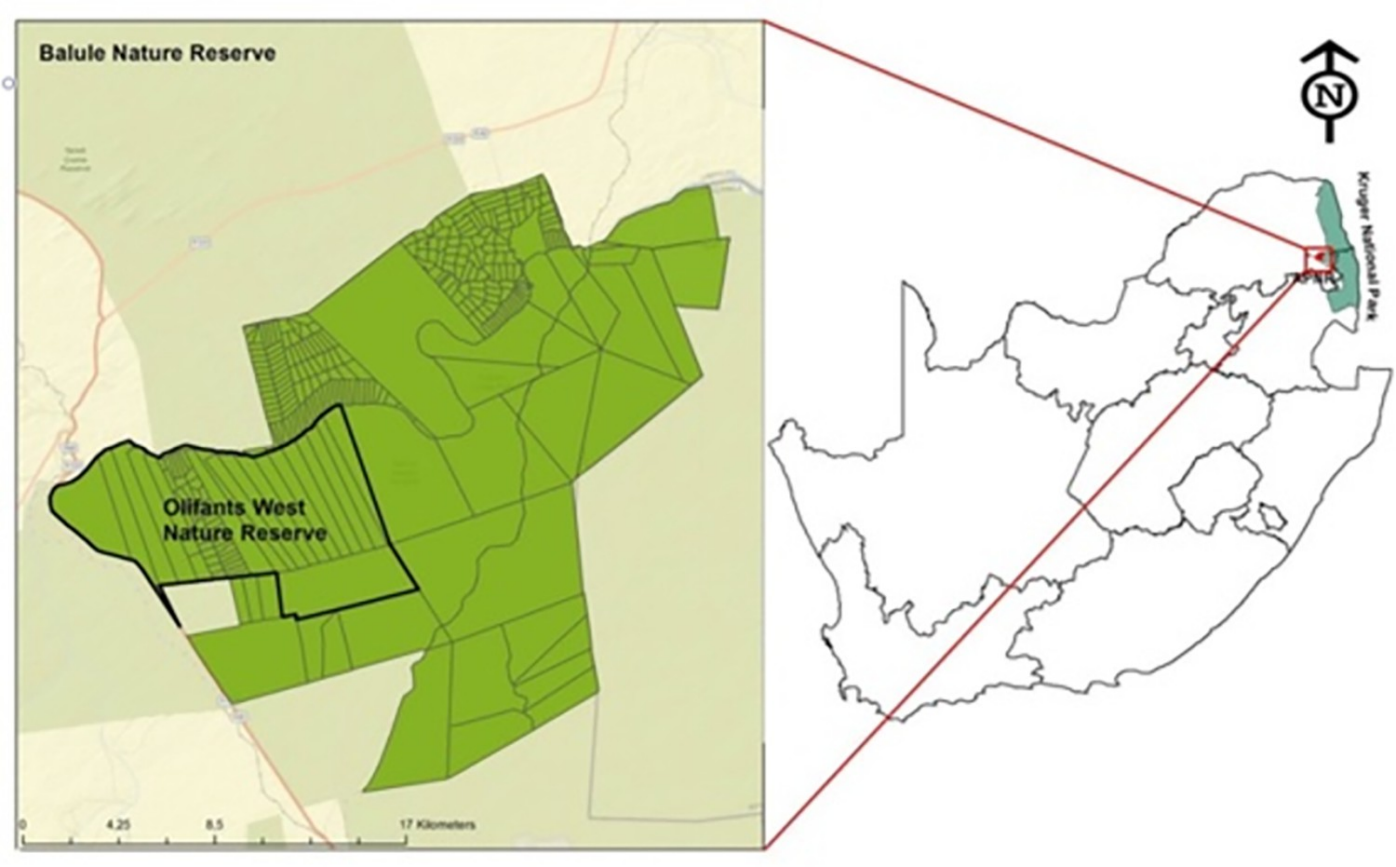
Location of the Balule Private Nature Reserve in relation to the Kruger National Park within South Africa. (Image created by the researcher).

The OWNR is located within the Balule Private Nature Reserve, forming part of the larger Kruger National Park (KNP) (Coordinates: -24.1987, 30.9090) (Fig 1). The OWNR is approximately 8 800 hm^2^ in size, with open borders into the KNP to the East and South. The western boundary of the OWNR is fenced and represents the furthest point to the West that animals can travel. The Olifants River forms the northern boundary of the OWNR.

The altitude of the study area ranges from 338 m.a.s.l in the east to 360 m.a.s.l in the west. The study area is arid savannah [42, 43] with granitoid-based geology of the Swazian and Randian age group, derived from the Basement complex [42, 43]. The predominant soil types found in the study area are granitic- and gabbro-derived [42–44].

**Fig 2:**
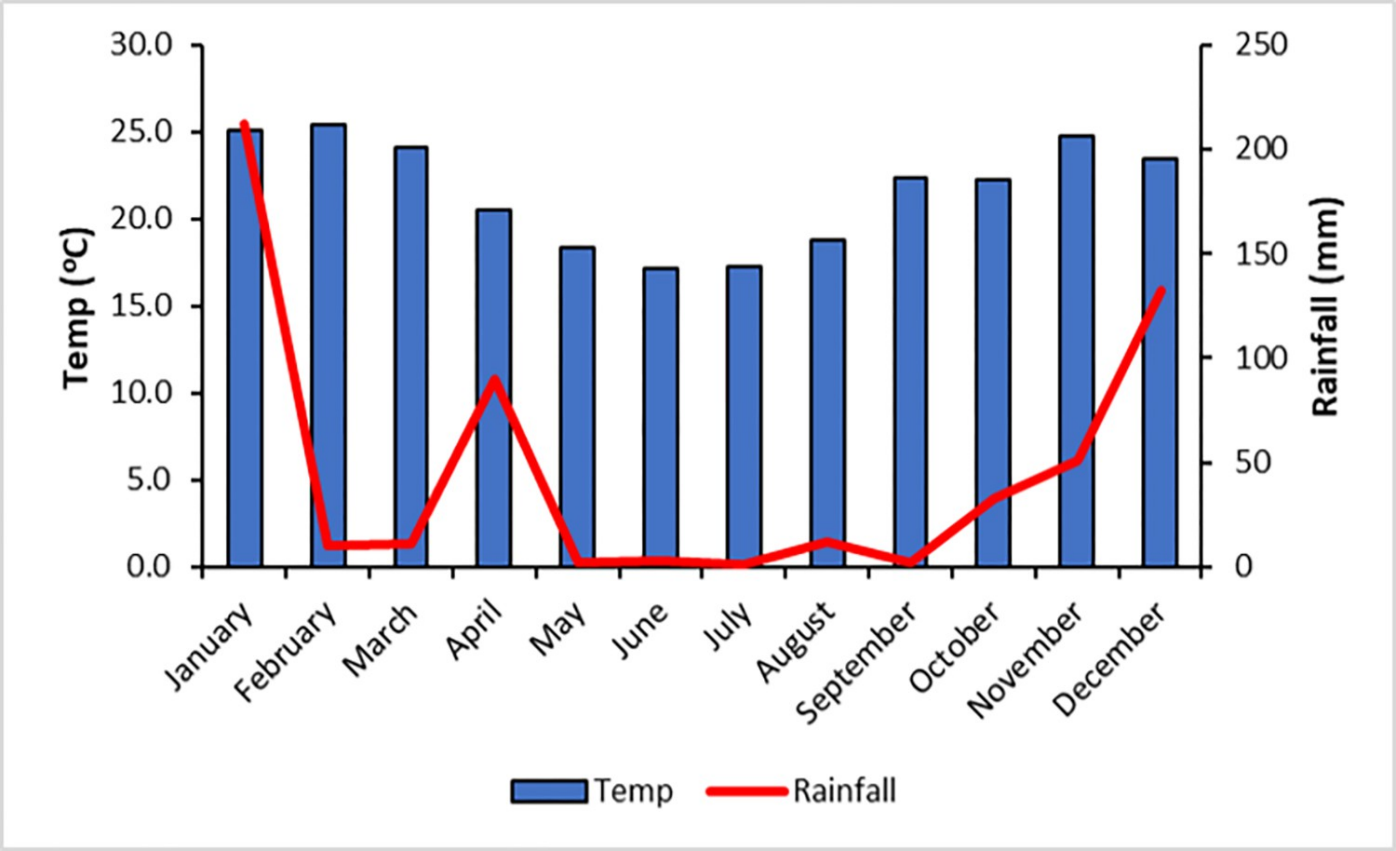
Temperature and rainfall data for the 2013 study period.

Climate data was collected from a weather station located at the OWNR research facility at the study site (Fig 2). Climate for the Savannah Biome is characterised by a clearly defined summer-rainfall pattern [45], with most of the rainfall occurring in the warm wet months between November and April. The cool dry months (May to October) are cooler and dryer, characterised by night-time temperatures that drop as low as 0°C in high altitude areas [45].

The OWNR is located entirely in the Savannah Biome characterised by a grass and forb dominated ground layer with an upper woody vegetation layer [45]. The vegetation is classified as belonging to the Granite Lowveld (SVI3) vegetation type [45].

### Waterhole selection

**Fig 3:**
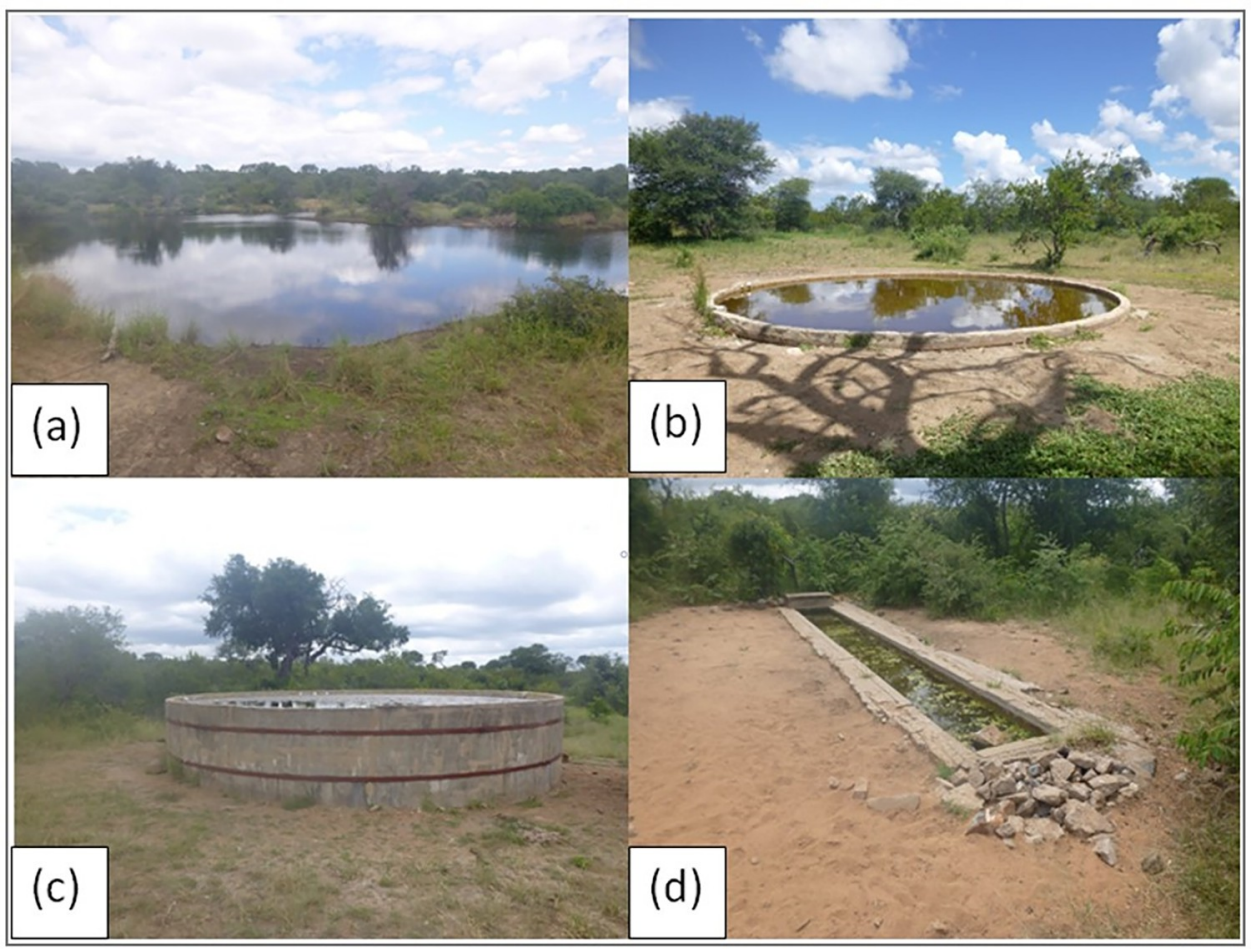
Examples of the different waterhole types at the study area. (a) is an example of an earth dam, (b) is an example of a pan, (c) is an example of a reservoir, and (d) is an example of a trough. (Photographs taken by the researcher).

Four artificial waterhole types were present at the study site and selected for this study namely, earth dams, concrete pans, reservoirs and troughs (Fig 3). These waterhole types were either already present or were established after the creation of the Balule Private Nature Reserve in the early 1990’s. Earth dams did not have a concrete base or sides and were established around natural features. Earth dams were in woody areas where there was no need for clearing bush to build concrete structures. Reservoirs had a concrete base with high sides and were placed close to boreholes in relatively dense woody vegetation. Concrete pans had a concrete base with low sides and were in relatively open areas dominated by grass. Troughs were often associated with reservoirs, had a concrete base and were usually placed some distance away from reservoirs in open areas dominated by grass. All waterhole types provided water perennially and were chosen based on their spatial orientation and ease of access from the road network in the reserve.

Eleven waterholes (Table 1) were monitored for the wet (November to April) and dry (May to October) seasons. Days were split into four daily periods constituting night (18:01 to 06:00), morning (06:01 to 10:00), midday (10:01 to 14:00) and afternoon (14:01 to 18:00).

**Table 1:** Olifants West Nature Reserve artificial waterhole types with their local names, waterhole type, capacity, size categories and number of camera traps placed at the waterholes.

Artificial Waterhole Characteristics				
Name	Type	Capacity (m^3^)	Size category	Number of camera traps
Ngala	Earth dam	100–1 000	Medium	1
Oxford big dam	Earth dam	> 1 000	Large	2/3 *
Singwe big dam	Earth dam	> 1 000	Large	3/4 *
Leopard’s view	Concrete pan	< 100	Small	1
Singwe bush camp	Concrete pan	< 100	Small	1
Toni’s dam	Concrete pan	< 100	Small	1
Nyala	Reservoir	100–1 000	Medium	1
Nzulwini	Reservoir	100–1 000	Medium	1
Van Wyk’s	Reservoir	100–1 000	Medium	1
Nyala	Trough	< 100	Small	1
Van Wyk’s	Trough	< 100	Small	1

* Number of camera traps deployed at Singwe Big Dam and Oxford big dam varied depending on water surface area, which was affected by rainfall.

### Waterhole monitoring

Camera traps were used to monitor the selected waterholes 24 hours a day, seven days a week, from January to December 2013. Camera traps used included Bushnell (model 119466), Tasco (model 9215), Scoutguard (model SG550) and Reconyx Rapidfire (RC55). All camera brands had similar specifications and were the models available for use by the researcher. Camera settings were standardised for all camera types to take five megapixel photographs every 1.2 seconds once movement was detected. All cameras could take infrared photos at night.

The cameras were placed in the field for a two-week period prior to starting with the data collection to ensure that the correct camera settings and angles were used, and to ensure that all batteries, cameras and SD cards were functional. Doing this could have acclimatised the various species visiting the waterholes to the presence of the cameras as the researcher did not observe any avoidance behaviour prior to, or during the study when time was spent at the various waterholes. Camera traps were checked every seven days for damage, to download imagery, clear memory cards, replace batteries, and to ensure that they were still properly positioned. Data collected from the camera traps provided information about the number of days each waterhole was effectively photographed.

Camera traps were positioned to maximise the percentage of waterhole edge covered, with additional camera traps used to cover larger waterholes. For waterholes that had more than one camera trap, dates and times on the various camera traps were synchronised so that when viewing images from various cameras, it was possible to identify the same animals i.e. images of animals taken within a few minutes of each other on different cameras could be isolated and the animals identified as being the same ones. Also, when there was more than one camera at a waterhole, the cameras were placed in such a way that there was slight overlap of their fields-of-view, resulting in animals moving from one camera’s field-of-view to another camera’s field-of-view being identified as the same animals.

Invariably, there were occasions when camera traps had to be removed for repairs or replacement. To cater for days when waterholes did not have cameras, a sightings-per-day value was calculated for all cameras by dividing the number of sightings at a waterhole by the number of days the waterhole was effectively monitored. This allowed for direct comparison of visitation data between waterhole types. The number of camera traps deployed at Singwe Big Dam and Oxford Big Dam varied depending on available surface water, which was influenced by rainfall. When these dams were very full, more camera traps were placed out than when the dams were empty and had less surface water.

Photographs from the camera traps were digitally dated and time-stamped for accuracy when doing comparisons. Information obtained from the camera traps included time of day that elephant, black rhino and white rhino utilised the various waterhole types, social grouping types frequenting the waterholes, number of animals, and the duration of stay at waterholes. Elephant group types identified were bachelors, bachelor groups and breeding herds. Black rhino group types were bachelors, bachelor groups, cows, cow groups, bull and cow groups and unknown adults. White rhino groups were the same as for black rhino, except for cow groups, which were not observed during this study as this group type likely used waterholes without camera traps.

A single visitation to a waterhole was defined as a single photograph or sequence of photographs isolated by a minimum of five minutes from any other photographs according to the timestamp. The five-minute interval is based on direct observations made by the researcher. The timings of visitations (time of day) and duration of stay by elephant and rhinos were used to determine waterhole preferences and utilisation patterns. Duration of stay at a waterhole was calculated by subtracting departure times from associated arrival times at waterholes.

### Data analyses

Binomial tests with confidence intervals were used to test for waterhole preference by the different species and their social group types [46]. The rationale for using a binomial test was that it is an exact test, providing a rigorous examination of the observed outcomes against expected probabilities for two distinct possible outcomes, selection (preference) or rejection (no preference) [46]. The precision of the binomial test is particularly beneficial when evaluating waterhole preferences, as it ensures accurate statistical inferences, even in scenarios with small sample sizes or discrete datasets.

The use of binomial tests in this study extends beyond a general preference assessment. By conducting tests across different seasons (wet and dry) and daily periods (night, morning, midday, and afternoon), we capture a comprehensive view of waterhole utilization patterns. Only significant results with a lower 95% confidence interval above the chance threshold of 25% were reported, ensuring that detected preferences were not due to chance. The 25% threshold was established based on the equal probability of selection among the four distinct waterhole types.

Additionally, a series of Poisson General Linear Models (GLM’s) were run to determine the impact of five predictor variables, Season (1 Wet or 2 Dry), Daily Period (1 Morning, 2 Midday, 3 Afternoon, 4 Night), Waterhole type (1 Earth Dam, 2 Concrete 3 Pan, 4 Reservoir, 5 Trough), Waterhole Size (1 Small, 2 Medium, 3 Large), and Social Group Type (1 Bachelor, 2 Bachelor group, 3 Cow, 4 Cow group, 5 Cow and calf, 6 Unknown adult, 7 Breeding herd, 8 Bull and cow) on the frequency of visits to waterholes by the three study species [47]. Poisson GLMs were chosen because the response variable represented count data, characterized by discrete, positive integer values, and this model type accounts for non-normality and variance that is not constant [47]. All predictor variables were treated as fixed effects, with no need to control for random effects, thus eliminating the necessity for generalized linear mixed models [47]. Grouping variables used in the GLM’s included Season, Daily Period and Waterhole Type.

To compare hourly visitation frequencies across all waterhole types for the three study species, the data were square root transformed to stabilize variance and improve the validity of analyses done [46].

All statistical tests were performed using IBM SPSS Statistics 23.

## Results

A total of 770 elephant observations (n = 378 wet season, n = 392 dry season), 107 black rhino observations (n = 73 wet season, n = 34 dry season) and 30 white rhino observations (n = 13 wet season, n = 17 dry season) were recorded by the camera traps during this study.

### Visitation frequency to waterholes

**Fig 4:**
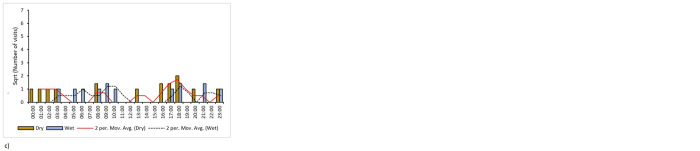
Visitation frequencies to all waterhole types across the hours of a day for the wet and dry seasons with a two-period moving average to show seasonal trends for a) Elephant, b) Black rhino, and c) White rhino.

Seasonal visitation frequencies to all waterhole types by elephants is shown in Fig 4A, for black rhino in Fig 4B and for white rhino in Fig 4C.

### Proportional visits to the different waterhole types

**Fig 5:**
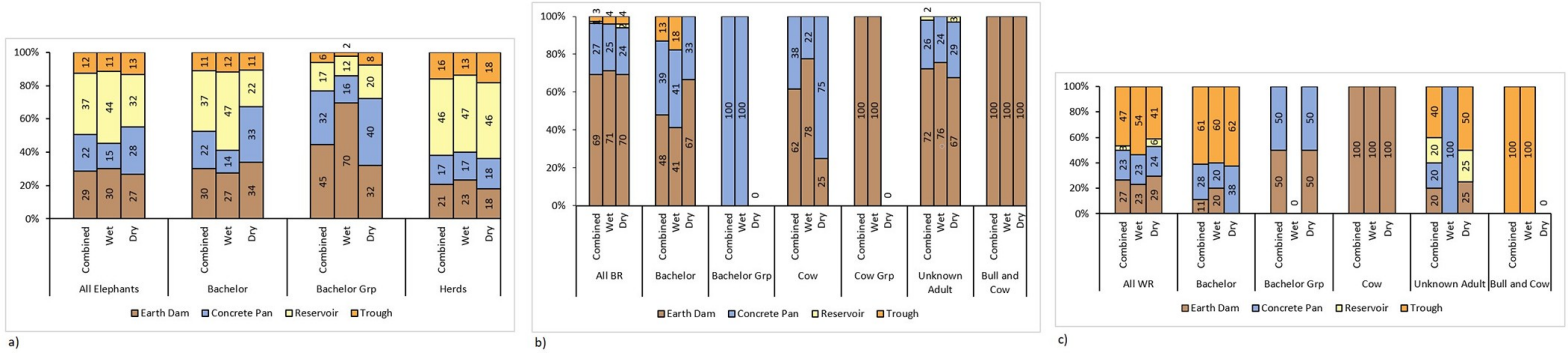
Combined wet and dry season, wet season and dry season proportional use of earth dams, concrete pans, reservoirs and troughs by a) all elephants, bachelors, bachelor groups and herds; b) all black rhinos, bachelors, bachelor groups, solitary cows, cow groups, unknown adults, and bull and cow groups; and c) all white rhinos, bachelors, bachelor groups, solitary cows, unknown adults and bull and cow groups.

Combined seasons, wet season and dry season proportions of visits to the different waterhole types by elephants are depicted in Fig 5A, black rhinos in Fig 5B, and white rhino in Fig 5C.

**Fig 6:**
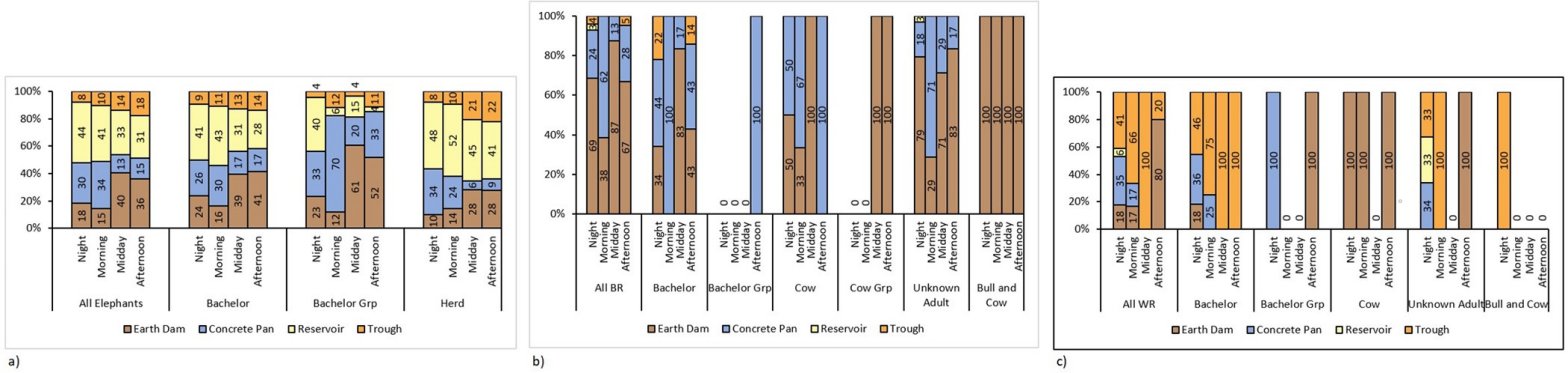
Night, morning, midday and afternoon period proportional use of earth dams, concrete pans, reservoirs and troughs by a) all elephants, bachelors, bachelor groups and herds, b) all black rhinos, bachelors, bachelor groups, solitary cows, cow groups, unknown adults and bull and cow groups, and c) all white rhinos, bachelors, bachelor groups, solitary cows, unknown adults and bull and cow groups.

Daily period (night, morning, midday and afternoon) proportions of visits to the various waterhole types by the different elephant social group types are depicted in Fig 6A, black rhino social group types in Fig 6B, and white rhino social group types in Fig 6C.

### Waterhole type preferences

Elephant binomial test results indicating seasonal and daily-period waterhole type preferences by the different elephant social group types are shown in Table 2.

**Table 2:** Elephant binomial test results indicating seasonal and daily-period waterhole type preferences by the different elephant social group types. Only results indicating a preference (lower 95% CI greater than 25% and significant) are shown.

Elephant						
Group type	Season and Daily Period	Preferred waterhole type	95% CI lower	95% CI upper	Z-Statistic	*P*
All	Comb Wet & Dry	Reservoirs	33.72	40.66	7.78	< 0.001
	Wet	Reservoirs	37.81	48.02	8.02	< 0.001
	Dry	Reservoirs	27.05	36.49	3.03	0.002
	Night	Reservoirs	37.79	50.13	7.06	< 0.001
	Morning	Reservoirs	30.72	54.14	3.54	< 0.001
	Midday	Earth dams	33.82	49.98	5.30	< 0.001
	Afternoon	Earth dams	29.13	43.37	3.47	< 0.001
Bachelor	Comb Wet & Dry	Reservoirs	30.77	41.86	4.50	< 0.001
	Wet	Reservoirs	39.45	54.83	6.69	< 0.001
	Dry	Earth dams	26.07	42.84	2.41	0.016
	Night	Reservoirs	32.70	50.15	4.29	< 0.001
	Morning	Reservoirs	27.10	60.51	2.56	0.010
	Midday	Earth dams	28.04	51.75	2.81	0.005
	Afternoon	Earth dams	29.44	54.44	3.08	0.002
Bachelor Grps	Comb Wet & Dry	Earth dams	35.75	53.22	5.16	< 0.001
	Wet	Earth dams	53.88	82.82	6.78	< 0.001
	Dry	Concrete pans	29.81	50.78	3.29	0.001
	Morning	Concrete pans	44.04	89.69	4.34	< 0.001
	Midday	Earth dams	47.44	73.45	6.39	< 0.001
	Afternoon	Earth dams	31.95	71.33	3.22	0.001
Breeding herds	Comb Wet & Dry	Reservoirs	40.50	51.57	8.83	< 0.001
	Wet	Reservoirs	38.79	54.60	6.38	< 0.001
	Dry	Reservoirs	37.65	53.09	6.11	< 0.001
	Night	Reservoirs	38.44	58.67	5.46	< 0.001
	Morning	Reservoirs	36.42	68.00	4.10	< 0.001
	Midday	Reservoirs	34.63	55.28	4.48	< 0.001
	Afternoon	Reservoirs	31.41	52.12	3.69	< 0.001

Binomial test results showing seasonal and daily-period waterhole type preferences by the different black rhino social group types are depicted in Table 3.

**Table 3:** Binomial test results indicating seasonal and daily-period waterhole type preferences by the different black rhino social group types. Only results indicating a preference (lower 95% CI greater than 25% and significant) are shown.

Black rhino						
Group type	Season and Daily Period	Preferred waterhole type	95% CI lower	95% CI upper	Z-Statistic	*P*
All	Comb Wet & Dry	Earth dams	59.50	77.73	10.55	< 0.001
	Wet	Earth dams	59.44	81.23	9.12	< 0.001
	Dry	Earth dams	46.49	80.26	5.35	< 0.001
	Night	Earth dams	54.59	81.75	7.18	< 0.001
	Morning	Concrete pans	31.58	86.14	3.04	0.002
	Midday	Earth dams	67.64	97.34	7.07	< 0.001
	Afternoon	Earth dams	43.04	85.41	4.41	< 0.001
Bachelor	Comb Wet & Dry	Earth dams	26.80	69.39	2.53	0.012
	Midday	Earth dams	35.87	99.58	9.92	< 0.001
Cow	Comb Wet & Dry	Earth dams	31.54	86.12	3.04	0.002
	Wet	Earth dams	39.99	97.19	3.66	0.000
	Dry	Concrete pans	28.64	98.14	8.88	< 0.001
	Midday	Earth dams	47.82	100.00	10.95	< 0.001
Cow Group	Comb Wet & Dry	Earth dams	29.24	100.00	3.00	0.003
	Wet	Earth dams	29.24	100.00	3.00	0.003
Unknown adult	Comb Wet & Dry	Earth dams	58.35	83.54	8.01	< 0.001
	Wet	Earth dams	57.79	88.94	6.74	< 0.001
	Dry	Earth dams	43.07	85.44	14.66	< 0.001
	Night	Earth dams	62.10	91.30	22.44	< 0.001
	Morning	Concrete pans	29.02	96.32	9.10	< 0.001
	Midday	Earth dams	29.02	96.32	9.10	< 0.001
	Afternoon	Earth dams	35.87	99.58	9.92	< 0.001
Bull & cow	Comb Wet & Dry	Earth dams	73.54	100.00	6.00	< 0.001
	Wet	Earth dams	66.37	100.00	5.20	< 0.001
	Dry	Earth dams	29.24	100.00	8.49	< 0.001
	Midday	Earth dams	54.07	100.00	12.00	< 0.001
	Afternoon	Earth dams	39.76	100.00	9.80	< 0.001

Table 4 shows binomial test results to determine preferences for the waterhole types by the different white rhino social group types.

**Table 4:** Binomial test results indicating seasonal and daily-period waterhole type preferences by the different white rhino group types. Only results indicating a preference are shown (lower 95% CI greater than 25% and significant).

White rhino						
Group type	Season and Daily Period	Preferred waterhole type	95% CI lower	95% CI upper	Z-Statistic	*P*
All	Comb Wet & Dry	Troughs	28.34	65.68	2.74	0.010
	Wet	Troughs	25.14	80.78	2.40	0.016
	Afternoon	Earth dams	28.36	99.59	2.84	0.005
Bachelor	Comb Wet & Dry	Troughs	35.73	82.69	12.36	< 0.001
	Wet	Troughs	26.24	87.84	9.04	< 0.001
Cow	Comb Wet & Dry	Earth dams	39.76	100.00	9.80	< 0.001
	Dry	Earth dams	29.24	100.00	8.49	< 0.001

### Effects of season, daily period, waterhole size, waterhole type and social group type on frequency of visits

GLM results for the most significant models with the best fit for the three study species are shown in Table 5.

**Table 5:** Results for a series of GLM’s showing the models with the best fit for White rhino, Black rhino and Elephant.

Coefficients	*β*	Z value	CI	Pr(>|z|)
**White Rhino**				
Afternoon Period (DailyPeriod*GroupType)	1.003	-1.977	-3.95:1.94	0.05
Dry Season:Afternoon Period (Season*DailyPeriod)	2.043	2.516	0.45:3.63	0.01
**Black Rhino**				
Unknown Adults (WaterholeType*GroupType)	1.229	3.640	0.57:1.89	< 0.00
Unknown Adults (Period*GroupType)	1.269	3.362	0.53:2.01	< 0.00
Large Waterholes (DailyPeriod*WaterholeSize)	0.700	2.040	0.03:1.37	0.04
Unknown Adults (Season*GroupType)	1.253	2.706	0.35:2.16	0.01
**Elephant**				
Medium Waterholes (WaterholeSize*GroupType)	0.401	3.046	0.14:0.66	< 0.00
Reservoir (WaterholeTyppe*GroupType)	0.706	5.042	0.43:0.98	< 0.00
Reservoir:Breeding Herd (WaterholeTyppe*GroupType)	0.676	3.379	0.28:1.07	< 0.00
Trough:Breeding Herd (WaterholeTyppe*GroupType)	0.978	3.533	0.44:1.52	< 0.00
Morning Period:Bachelor Groups (Period*GroupType)	1.028	2.859	0.32:1.73	< 0.00
Midday Period:Bachelor Groups (Period*GroupType)	1.247	4.668	0.72:1.77	< 0.00
Medium Waterholes (Period*WaterholeSize)	0.303	2.253	0.04:0.57	0.02
Midday Period:Large Waterholes (Period*WaterholeSize)	1.131	4.202	0.60:1.66	<0.00
Afternoon Period:Large Waterholes (Period*WaterholeSize)	1.090	3.947	0.55:1.63	< 0.00
Midday Period (Period*WaterholeType)	0.490	2.761	0.14:0.84	0.01
Concrete Pan (Period*WaterholeType)	0.996	5.431	0.64:1.36	< 0.00
Reservoir (Period*WaterholeType)	1.385	8.057	1.05:1.72	< 0.00
Wet Season (Season*GroupType)	0.319	2.755	0.09:0.55	0.01
Breeding Herd (Season*GroupType)	0.253	2.181	0.03:0.48	0.03
Wet Season:Medium Waterholes (Season*WateholeSize)	0.549	3.266	0.22:0.88	< 0.00
Concrete pan (Season*WaterholeType)	0.515	3.784	0.25:0.78	<0.00
Reservoir (Season*WaterholeType)	0.626	4.719	0.37:0.89	< 0.00
Wet Season:Reservoir (Season*WaterholeType)	0.408	2.279	0.06:0.76	0.02

β = beta value, CI = Confidence Interval, Pr(>|z|) = significance

## Discussion

Elephants at OWNR consistently visited waterholes throughout the day, particularly between 09:00 and 20:00 during the dry season, and between 09:00 and 21:00 during the wet season (Fig 4A). These patterns align with another study conducted in the southern region of Kruger National Park [48]. The midday period, particularly around 11:00 to 12:00, emerged as the time of highest visitation to waterholes, coinciding with peak evapotranspiration and the need for cooling [17]. This finding is comparable to that of Hayward & Hayward [21] for their study across various parks and reserves including Kruger National Park, Pilanesberg National Park, Madikwe Game Reserve, Tembe Elephant Park and Botswana’s Mashatu Game Reserve. In the OWNR daily temperatures are generally high throughout the day, which is common in the lowveld of southern Africa, and only decreases in the evenings. Findings for a study done in Hwange National Park, Zimbabwe [20] indicated peak utilisation of waterholes by elephant at around 20:00, which matches a secondary peak we observed for the wet season. Limited surface water availability in Hwange National Park [20] could be a reason for elephants utilising waterholes less frequently and likely having a greater dependence on alternative thermoregulatory methods [17]. While elephants are generally considered to have no preference for drinking at a specific time [33], our findings suggest that probably due to their large body mass and associated water loss during hotter parts of the day, elephants tend to visit waterholes more frequently between late morning and early evening (09:00 and ~21:00).

In this study, elephants exhibited a clear preference for reservoirs throughout the year. This is likely because reservoirs have high concrete sides and bottoms, are filled with borehole water, and are often out of reach to other game species that could pollute the water. Other waterhole types are more likely to be contaminated with faecal matter and associated bacteria [23]. The avoidance of surface water with high bacteria levels has been substantiated by studies showing that elephants will rather dig for water to drink than use polluted surface water [49, 50]. This preference differs from the broader findings of Du Toit [33], who indicated an overall preference for natural waterhole types like earth dams. We did however find that elephants at the study site used earth dams at midday and in the afternoon for bathing or lying down in water to cool down during the hot daily temperatures of the wet season [32]. Reservoirs were preferred at night and in the morning, and earth dams at midday and in the afternoon (Table 2).

The behaviour of bachelor elephant groups differed from that of breeding herds, indicating potential social dynamics. Bachelor elephants preferred reservoirs in the wet season and earth dams in the dry season. Bachelor groups preferred earth dams in the wet season and concrete pans in the dry season. Breeding herds consistently preferred reservoirs throughout both seasons across all daily periods (Figs 5A and 6A). This suggests that female herd members may not tolerate bachelor groups, aligning with documented behaviour in the literature [34, 51]. Bachelor groups, composed of young males testing their strength, could pose a risk of injury to young elephants within herds [32, 34, 51]. Solitary mature bachelor elephants often linger near herds, waiting for mating opportunities with herd cows [34]. These lone bachelors showed a noticeable preference for reservoirs at certain times of the year and day (Table 2), roughly coinciding with the times that herds visited reservoirs [32, 34]. Although reservoirs were visited mostly at night, test results indicated no preference for a particular waterhole type at night (Table 2). Concrete pans were preferred in the morning, while earth dams were favoured at midday and in the afternoon.

The results of the generalized linear models (GLMs) to determine the effects of Season, Daily Period, Waterhole Size, Waterhole Type and social Group Type visiting waterholes on frequency of elephant visits to the waterholes revealed significant effects for various of these factors (Table 5). The main waterhole types selected at the study site were reservoirs and concrete pans. Waterholes were used most during the wet season, with a preference for medium sized waterholes. Breeding herds were the most frequently observed group visiting waterholes, favouring medium sized reservoirs and their troughs (Table 1). Adult and sub-adult animals that could reach water in reservoirs used these, while juvenile animals used troughs. Bachelor groups were commonly observed at waterholes during morning and afternoon periods. Overall, waterholes were most visited during midday, with reservoirs and concrete pans being the most frequented. Medium-sized waterholes were preferred across all daily periods, with large waterholes being used during midday and afternoon. Breeding herds, consisting of females and their young, may prioritize safety and the availability of clean water sources, leading to a consistent preference for reservoirs. Bachelor groups, composed of young males, may select waterhole types based on factors such as opportunities for interaction with breeding herds or their own social dynamics.

Black rhinos displayed different patterns in their waterhole visits compared to elephants. They visited waterholes across all daily periods, with specific peaks during the dry and wet seasons. Dry season peaks were between 02:00 and 03:00, at 06:00, between 13:00 and 15:00, and again at 21:00 (Fig 4B). Wet season peaks were at 10:00, 13:00, and again at 18:00 (Fig 4B). These results align with the findings of Du Toit [33], who found that black rhino drink throughout the day and night depending on environmental conditions and water availability, though further research is needed to clarify potential seasonal variations in this behaviour. They also align with previous studies conducted in different arid and semi-arid areas [52, 53]. In arid areas black rhino drink when it is cooler, often in the evenings or during the day on overcast days [52, 53]. Kasiringua, Kopij, & Procheş [52] reported that for the arid Waterberg National Park in Namibia, black rhino drink at night during the dry season where these authors classified them as evening and night drinkers. Seidel et al. [53] found that for the semi-arid Etosha National Park in Botswana, black rhino visited waterholes after dusk and before dawn. OWNR is not classified as an arid environment with extreme daily temperatures, resulting in the black rhino on the reserve visiting waterholes throughout the day and night, but with peaks as listed above.

Findings for this study indicate that black rhinos exhibited a preference for earth dams throughout the year (Fig 5B). Du Toit [33] reported a preference for artificial waterhole types, although the specific types were not detailed. Earth dams were favoured at night, midday, and in the afternoon, while concrete pans were preferred in the morning (Fig 6B and Table 3). Bachelor black rhino’s showed a preference for earth dams in the dry season and no preference for any waterhole type in the wet season (Fig 5B). They preferred this waterhole type during the midday period, with no preference during other daily periods. Even though black rhino bachelor groups visited concrete pans in the wet season during the afternoon period, binomial test results (Table 3) did not indicate a preference for this waterhole type. There were no recorded visits to any of the waterhole types for the dry season, leading us to believe that bachelor groups visited alternative water sources that did not have camera traps monitoring them. Solitary black rhino cows preferred earth dams in the wet season and concrete pans in the dry season. There was a preference for earth dams during the midday period, and although they visited concrete pans in the morning and afternoon, binomial test results did not indicate a preference for this waterhole type (Table 3). Black rhino cow groups favoured earth dams in the wet season, with no visits recorded for the dry season. As for black rhino bachelor groups, we speculate that black rhino cow groups visited alternative water sources that did not have camera traps monitoring them. While there were visits to earth dams during the midday and afternoon periods, and results were significant, lower confidence intervals were below the 25% chance threshold. There were no recorded visits to waterholes by cow groups during night and morning periods. The “unknown adult black rhino” category preferred earth dams across both the wet and dry seasons (Fig 5B). Earth dams were favoured at night, midday and in the afternoon, with concrete pans preferred in the morning (Fig 6B). Black rhino bull and cow groups preferred earth dams across both the wet and dry seasons and across all daily periods. We speculate that bachelor black rhino groups at the study site, often consisting of sub-adult bulls, used concreate pans to avoid confrontation with aggressive territorial bulls who were often with cows at earth dams. GLM results (Table 5) suggest that unknown black rhino adults preferred large (P = 0.04) earth dams (P < 0.00) in both the wet and dry seasons (P = 0.01) for three of the daily periods, midday, afternoon and night (P < 0.00).

White rhinos displayed specific patterns in their waterhole visits, with peaks during certain daily periods and variations between the wet and dry seasons. White rhino visits during the dry season peaked at 08:00 and between 16:00 and 18:00 (Fig 4C). Sutherland, Ndlovu, & Pérez-Rodrguez [48] recorded peaks in the dry season from 00:00 to 01:00, from 11:00 to 12:00 and a spike in visits between 17:00 and 22:00. Wet season waterhole visits for our study peaked at 09:00, 18:00 and again at 21:00. Wet season visits for the Sutherland, Ndlovu, & Pérez-Rodrguez [48] study showed a peak at 17:00 and from 20:00 to 22:00. Du Toit [33] states that white rhinos visit waterholes in the morning, at midday and in the afternoon. Kasiringua, Kopij, & Procheş [52], for the arid Waterberg National Park in Namibia, classified white rhino as evening and night drinkers. In general, our study has similar results to that of Sutherland, Ndlovu, & Pérez-Rodrguez [49] for the wet season, in that there are three peaks (Fig 4C) and differs from Du Toit [33] in that we recorded night visits (Fig 6C). Our results also differ from those of Kasiringua, Kopij, & Procheş [52], in that we recorded morning visits (Fig 6C).

The sample size for white rhino in this study was restricted due to lack of suitable habitat at the study site, compared to the elusive black rhino that prefers dense thickets. Although white rhino visited troughs during both the wet and the dry seasons (Fig 5C), binomial test results only indicate a preference for this waterhole type during the wet season (Table 4). Du Toit [33] found that white rhino had no preference for a particular waterhole type, whereas we found a preference for troughs and earth dams. Earth dams were preferred during the afternoon, and even though troughs were frequented at night, in the morning and at midday, results did not indicate a preference for troughs during these periods (Table 4). Bachelor white rhino preferred troughs across both the wet and dry seasons, and although troughs were visited across all daily periods, binomial test results did not indicate a preference for them, or any other waterhole type (Table 4). Bachelor white rhino groups visited earth dams and concrete pans during the dry season, with no visits recorded for the wet season. Test results showed no seasonal or daily period preferences for any waterhole types by bachelor groups (Table 4). Cows visited only earth dams across the wet and dry seasons (Fig 5C). Earth dams were visited by cows during the night, morning and afternoon periods, with no recorded visits at midday. Cows showed a preference for earth dams in the dry season. Unknown adults mostly visited troughs during the dry season and concrete pans during the wet season (Fig 5C). Concrete pans were visited at night, troughs in the morning and earth dams in the afternoon (Fig 6C). There were no recorded visits during the midday period. The unknown adult category showed no seasonal or daily preferences for any of the waterhole types. The bull and cow category visited troughs most during the combined wet and dry seasons, and during the wet season. Troughs were visited most at night with no recorded visits to waterholes for the other daily periods (Fig 6C). Binomial tests results for bull and cow groups showed no seasonal or daily preferences for any of the waterhole types. No visits to waterholes were recorded for the dry season, or the morning, midday and afternoon periods. As for black rhino, we believe that white rhino bachelor groups consisting primarily of sub-adults, avoided the waterhole type (troughs) that territorial bulls frequented with cows. Territorial bulls are aggressive and do not tolerate other bulls within their territories [32, 34].

GLM results for white rhino (Table 5), indicate that the interaction between the dry season and the afternoon period (P = 0.01) led to an increase in frequency of visits to waterholes, and that White rhino in the study area visited waterholes most during the afternoon period (P = 0.05). White rhino frequent open grassland areas and avoid areas with high densities of woody plants species, selecting the predominant waterhole types found in these areas, which are troughs and earth dams [32, 34].

Simultaneous use of waterholes by young elephant bachelor groups, black rhinos and white rhinos in areas where there are no large adult elephant bulls has been problematic in the past [51]. Several white rhino deaths were recorded in Pilanesberg National Park when young elephant bulls intimidated rhinos at waterholes in episodes of what were observed as young bachelor elephants trying to mate with rhinos [51]. This behaviour stopped as soon as large elephant bulls were introduced into the area [51, 54]. In terms of our findings that indicate both elephant bachelor groups and rhinos visit and prefer earth dams and concrete pans, it is important to ensure that a proper mature elephant bull hierarchy is in place for areas that contain bachelor groups of elephants and rhinos.

Although there was spatial and temporal overlap at waterholes in this study, the interaction between the three study species at the different waterhole types for OWNR was limited due to their various social groupings preferring different waterhole types at different times of the day. Elephants spent 37% of their time at reservoirs (Fig 5A) and had exclusive use of this waterhole type as neither of the rhino species could access water in reservoirs. Black rhino spent 69% of their time at earth dams (Fig 5B), which could be related to these waterholes being in relatively woody areas, which are the preferred habitat for black rhinos [32, 34]. White rhino spent 47% of their time at troughs (Fig 5C), suggesting that they, like the black rhino, prefer the waterhole types placed within their preferred habitat, which is relatively open areas dominated by grass. Both black and white rhino are territorial, which resulted in sub-adult bachelor groups using waterholes at different times than bull and cow groups, to prevent confrontation.

The differences in waterhole preferences between elephants and rhinos can be attributed to their distinct ecological and physiological characteristics. Elephants, with their large body size and high water requirements, are more inclined to select waterholes that provide clean water and opportunities for cooling and bathing. The preference for reservoirs may also be influenced by the presence of other game species that inhabit natural waterholes, making reservoirs a more appealing option for elephants seeking undisturbed water sources. On the other hand, black rhinos, known for their ability to survive in arid and semi-arid environments, exhibited a consistent preference for earth dams across seasons. This preference may be due to factors such as the availability of water and the avoidance of potential competitors or predators that frequent other waterhole types. White rhinos, being grazers and relying on open grasslands, showed a preference for troughs during the dry season, likely due to the concentrated availability of water in these artificial structures.

## Conclusion

Artificial waterholes play an important role in supporting wildlife populations in conservation areas [55]. This study investigated the preferences of elephants, black rhinos, and white rhinos for various artificial waterhole types in the South African savannah. Four waterhole types were examined: earth dams, concrete pans, reservoirs, and troughs. Findings revealed that each species exhibits distinct preferences and utilization patterns. Elephants favoured reservoirs, earth dams, and concrete pans, with breeding herds showing a particular preference for reservoirs and troughs. Black rhinos preferred large waterholes like earth dams, while white rhinos showed preferences for troughs and earth dams.

Understanding the preferences of the study species is important for effective waterhole management and conservation. By identifying the specific waterhole preferences of these species, conservationists and reserve managers can optimize the design and placement of waterholes, reducing competition and promoting the well-being of both target and non-target species [20, 35, 36]. Identifying which species prefer which waterhole types can help prevent conflicts and inform strategic waterhole placement [12, 20].

As natural surface-water diminishes during the dry season, artificial waterholes become essential for the behaviour and survival of various species [3, 21, 35, 36]. Information from this study can guide water provision and translocation strategies for the study species. Effective management of artificial waterholes is key to enhancing habitat use and ensuring the long-term viability of species utilising these waterholes [51, 54]. Given the poaching pressures the three study species face throughout their range [28–31], implementing efficient conservation plans requires careful consideration of artificial waterhole preferences.

Future research should explore additional factors influencing waterhole selection, including water quality, anthropogenic effects, and seasonal variations. Broader studies across different ecosystems will further refine conservation strategies and enhance our understanding of the study species needs. Furthermore, investigating waterhole preferences in different ecosystems and regions will enhance our understanding of the study species and contribute to the development of effective conservation strategies.

## Supporting information

S1 Data Elephant and rhino dataset used in the research. (XLSX)

S1 Text Elephant and rhino dataset used in the research README. (TXT)
